# Behaviour change interventions addressing antibiotic treatment seeking behaviour for respiratory tract infections in primary care settings: A scoping review protocol

**DOI:** 10.12688/hrbopenres.13831.1

**Published:** 2024-07-01

**Authors:** Anthony Maher, Kevin Roche, Eimear C Morrissey, Andrew W Murphy, Greg Sheaf, Cristin Ryan, Gerry Molloy

**Affiliations:** 1 School of Psychology, University of Galway, Galway, County Galway, Ireland; 2 Centre for Health Research Methodology, School of Nursing and MIdwifery, University of Galway, Galway, County Galway, Ireland; 3 Health Research Board Primary Care Clinical Trials Network Ireland, University of Galway, Galway, Ireland; 4 The Library of Trinity College Dublin, Dublin, Ireland; 5 School of Pharmacy and Pharmaceutical Sciences Panoz Institute, Trinity College Dublin, Dublin, Ireland

**Keywords:** Antimicrobial stewardship; anti-bacterial agents; behavioural sciences; intervention design; primary care.

## Abstract

**Objective:**

This scoping review aims to synthesise the extent and type of evidence on behaviour change interventions which address antibiotic treatment seeking behaviour for respiratory tract infections in the primary care/community setting.

**Introduction:**

Antimicrobial Resistance is recognised as a global health and economic threat by the World Health Organization and World Bank. Several lines of evidence point to patient and public demand as a key driver of inappropriate antibiotic use. Current policy initiatives acknowledge the need to prepare for the future by managing public expectations regarding antibiotics, especially for influenza-like illness and other respiratory tract infections. These initiatives emphasise the importance of designing and evaluating effective interventions that generate actionable knowledge for policy and practices related to the appropriate use of antibiotics. Behaviour change interventions, in this context, can aim to modify patients' attitudes, beliefs, and behaviours regarding antibiotics.

**Inclusion criteria:**

Identified studies will describe behaviour change interventions aimed at potential patients/participants within the primary care/community setting that address patient expectations of antibiotic use for respiratory tract infections. Diagnoses for respiratory tract infections will be classified by ICD-10 criterion.

**Methods:**

This scoping review will search the literature in Medline, Embase, CINAHL, PsycINFO, Web of Science Core Collection, Scopus, and Google Scholar to explore behaviour change interventions used to reduce expectations of antibiotics for respiratory tract infections in primary care. This review will follow the Joanna Briggs Institute guidelines for scoping reviews. It will be reported according to the Preferred Reporting Items for Systematic reviews and Meta-Analyses extension for Scoping Reviews.

## Introduction

The World Health Organization (WHO) has declared the misuse and overuse of antimicrobials as one of the main drivers in the development of drug-resistant pathogens, accelerating the prevalence of antimicrobial resistance (AMR) ^1, 2^. It is estimated that up to 1.3 million fatalities are due to AMR each year, with a projected tenfold increase by 2050. As an ongoing and escalating critical global health issue, multi-sectoral action is required in order to combat this worldwide healthcare challenge ^2, 3^. The creation of actionable knowledge that can help reduce AMR by enhancing the appropriate use of antibiotics among the public is recognised as a pressing area for global health research ^4^. In particular, the role of antibiotic treatment seeking behaviour among the public and its subsequent influence on antibiotic prescribing practices in primary care, particularly for respiratory tract infections (RTIs), has been identified has a key area for further investigation ^3, 4^. Behaviour change interventions in this domain can be seen as a proactive approach in promoting prudent antibiotic use for RTIs in the community ^5. 6^.

2022 data and surveillance reports from the European Centre for Disease Prevention and Control (ECDC) show that community antimicrobial consumption has increased per capita compared to the preceding two years, namely 2020 and 2019 ^7^. Nevertheless, it continues to exhibit a downward trend over a ten-year period ^7, 8^. Other estimates indicate a increasing trend of sporadic broad spectrum antibiotic use across the ECDC’s states. This emphasises an increased need to avoid antibiotics that fall outside the WHO’s AWaRe classification of Access group antibiotics ^7^. This increase may be partly due to a rise in RTI diagnoses, as a result of reduced immunity brought about by decreased exposure to common pathogens during the early part of the SARS-COV-2 pandemic and the subsequent resumption of usual social activity ^9, 10^. These trends underscore the importance of addressing antimicrobial consumption trends and optimising antibiotic use to mitigate the impact of post-pandemic infectious diseases.

RTIs are known to represent one of the most common reasons for antibiotic prescriptions in primary care settings. Despite the majority of RTIs being viral in aetiology, antibiotics are frequently prescribed inappropriately, contributing to the development of antibiotic resistance ^11^. Studies have shown that antibiotics are prescribed in up to 60% of cases of acute respiratory infections, such as acute bronchitis, sinusitis, and pharyngitis, where antibiotics provide little to no benefit ^12, 13^. In these instances of overuse and misuse, complications such as increased healthcare expenditure, treatment failures and adverse drug effects can arise ^14^. Managing public expectations for the prescribing of antibiotics, particularly for influenza-like illnesses (ILI) and other RTIs, through effective behavioural science-based research aimed at managing AMR, is recognised by current international policy initiatives, as well as WHO AMR related surveillance reports ^15^.

The recognition of social and behavioural sciences' perspectives has led to the development, evaluation, and implementation of effective psychological interventions aimed at reducing antibiotic prescribing within the context of AMR management ^16– 18^. For instance, the utilisation of delayed prescriptions can counteract 'action bias' – defined as “an impulse to act in order to gain a sense of control over a situation and eliminate a problem ^19^.” The various theories underlying behavioural interventions have been distilled in a small number of broad domains to classify the key determinants of health-seeking behaviours ^19, 20^. For example, the COM-B model integrates Capability, Opportunity, and Motivation as the key domains driving behaviour. This framework has provided a theoretical lens for examining behaviour change interventions in primary care settings, aimed at addressing antibiotic prescribing behaviours among providers ^21– 24^. Moreover, alongside these 'supply-side' solutions, behaviour strategies are necessary to address and diminish public expectations and demands for antimicrobials ^25, 26^.

A recent systematic review of interventions reported on the improvement of antibiotic use patterns from behaviour change interventions (BCIs) within low- and middle-income countries, however these were not specifically addressing the context of RTIs ^27^. In recent years, there has been increasing focus on developing interventions for the often-underreported ‘demand-side’ of the antibiotic equation. For example, a randomised controlled trial (RCT) in the UK examined behaviour change approaches for reducing expectations for antibiotics in primary care ^28^. There has been limited attempts to synthesise these kinds of interventions, therefore a scoping review was identified as the most appropriate methodology to address this knowledge gap given the limited current background literature. To our knowledge, there is no other systematic or scoping review that looks to synthesise the available evidence on BCIs aimed at patients and applied locally within primary care and community settings aimed, to reduce antibiotic treatment seeking behaviour for RTIs.

## Review question

*This review aims to explore what BCIs are being used to lower patient and carer expectations for the provision of antibiotics related to RTIs, as diagnosed by ICD-10 criterion*.

*The objectives of the review are to:*

○ *Identify specific BCIs that have been applied and evaluated to reduce antibiotic treatment seeking behaviour for RTIs*.
○ *Characterise the attributes of the BCIs by mapping them to the COM-B model within the Behaviour Change Wheel' framework*.
○ *Explore if behaviour change theories are embedded within interventions that are used to reduce antibiotic treatment seeking behaviour for RTIs*.
○ *Explore what gaps exist presently in the literature that need to be addressed in future research for BCIs.*

## Eligibility criteria

### Participants

*Eligible studies will focus on the patient or carers within the community/primary healthcare setting, that physically or conceptually present with an RTI, as diagnosed by an ICD-10 criterion.*

### Concept

*An objective or self-reported change of an individual or public groups antibiotic seeking behaviour relating to RTIs – this may be set in the form of improved (or intention to improve) appropriate use, reduced consumption, increased knowledge, or improved attitude/belief. This excludes BCIs which target healthcare professionals only*.

### Context

Relevant publications from any country that focuses on primary or community care, will be included.

### Types of sources

This scoping review will consider both experimental and quasi-experimental study designs including RCTs, non-RCTs, pre-post studies and interrupted time-series studies. In addition, analytical observational studies including prospective and retrospective cohort studies, case-control studies and analytical cross-sectional studies will be considered for inclusion.

In addition, systematic reviews that meet the inclusion criteria will also be considered.

## Methods

The proposed scoping review will be conducted in accordance with the JBI methodology for scoping reviews ^29^. A preliminary search of MEDLINE, PROSPERO, the Cochrane Database of Systematic Reviews and JBI Evidence Synthesis was conducted and no current or underway systematic reviews or scoping reviews on the topic were identified. This scoping review protocol was registered on the Open Science Framework and is available at https://doi.org/10.17605/OSF.IO/DZF9C.

### Search strategy

*The search strategy will aim to locate both published and unpublished studies. An initial limited search of MEDLINE and CINAHL will be undertaken to identify articles on the topic. The text words contained in the titles and abstracts of relevant articles, and the index terms used to describe the articles will be used to develop a full search strategy for Medline, Embase, CINAHL, PsycINFO, Web of Science Core Collection, Scopus, and Google Scholar (see Appendix I). The search strategy, including all identified keywords and index terms, will be adapted for each included database and/or information source. The reference list of all included sources of evidence will be screened for additional studies*.

*Studies published in any language will be included. Studies published since 2000 will be included, given the timing at which the implementation of the theoretical domain’s framework took place across interventions*.

### Study/Source of evidence selection

*Following the search, all identified citations will be collated and uploaded into Covidence® and duplicates removed. Titles and abstracts will then be screened by two or more independent reviewers for assessment against the inclusion criteria for the review. Potentially relevant sources will be retrieved in full, and their citation details imported into the JBI System for the Unified Management, Assessment and Review of Information (JBI SUMARI) (JBI, Adelaide, Australia) ^25^. At least 10% of full text of selected citations will be assessed in detail against the inclusion criteria by two or more independent reviewers. One independent reviewer will complete remaining screening of full text articles. Reasons for exclusion of sources of evidence at full text that do not meet the inclusion criteria will be recorded and reported. Any disagreements that arise between the reviewers at each stage of the selection process will be resolved through discussion, or with an additional reviewer/s. The results of the search and the study inclusion process will be reported in full in the final scoping review and presented in a Preferred Reporting Items for Systematic Reviews and Meta-analyses extension for scoping review (PRISMA-ScR) flow diagram) ^30^*.

### Data extraction

*Data will be extracted from at least 10% of included papers by two or more independent reviewers using a data extraction tool developed by the reviewers, with inter-rater reliability results tabulated and reported. The data extracted will include specific details about the participants, concept, context, study methods and key findings relevant to the review question/s*.

*A draft extraction form is provided (see Table 1). The draft data extraction tool will be modified and revised as necessary during the process of extracting data from each included evidence source. Modifications will be detailed in the results paper. Any disagreements that arise between the reviewers will be resolved through discussion, or with an additional reviewer/s. If appropriate, authors of papers will be contacted to request missing or additional data, where required*.

**Table 1.** Sample tool to be used for data extraction.

Author, Year, Country	Aim	Type of evidence source ( *e.g*. research report/ policy document)	Study design (+/- control group)	Population (Description of target)	Concept (Type of BCI to inform theoretical framework)	Context (Setting/ HCPs involved/ technology)	Description of BCI used ( *e.g.* technique, purpose)	Outcomes measured (effect/ impact – primary, secondary)
								
								
								
								

BCI = Behaviour Change Intervention HCP = Health Care Professional

### Data analysis and presentation

*The mapped data will be analysed to identify patterns, trends, and relationships between the characteristics of BCIs and the domains of the COM-B-model. Common themes and concepts will be synthesised to provide a comprehensive understanding of how the interventions align with the model*.

*The presentation of the scoping review will include a synthesis in the form of a narrative summary. This will accompany the tabulated and/or charted results and will describe how the results relate to the reviews objective and question/s. of the findings, including key themes, trends, and recommendations for future research and practice*.

## Data Availability

No data are associated with this article.

## Appendices

### Appendix I: Draft Search strategy

(((Respiratory OR chest) NEAR/2 (disease* OR infection*)) OR "RTI*" OR Bronchitis OR "Common cold*" OR Croup OR Epiglottitis OR Flu OR Influenza* OR Laryngitis OR Laryngotracheitis OR Otalgia OR Pharyngitis OR Pleurisy OR Pneumo* OR Rhinitis OR Sinusitis OR Tonsillitis OR Breathless* OR Congestion OR Cough* OR Earache* OR Headache* OR "High temperature*" OR "Muscle ache*" OR "Runny nose*" OR Sneez* OR "Sore throat*" OR Wheez*)

AND

(((Family OR General) NEAR/2 (doctor* OR physician* OR pract*)) OR ((Community OR Primary) NEAR/2 (care OR center* OR centre* OR healthcare)) OR Ambulatory OR Pharmac* OR (Home NEAR/2 visit*))

AND

((antibiotic* OR "anti biotic*" OR antimicrob* OR "anti microb*") NEAR/5 (resistan* OR prescrib*))

AND

(accept* OR access* OR approach* OR availab* OR behav* OR chang* OR communicat* OR convenien* OR decision* OR engag* OR guid* OR influen* OR intention* OR interven* OR motiv* OR nudg* OR persua* OR plan* OR polic* OR procedure* OR program* OR strateg* OR support* OR train* OR uptake*)
