# Behaviour change interventions addressing antibiotic treatment seeking behaviour for respiratory tract infections in primary care settings: A scoping review protocol

**DOI:** 10.12688/hrbopenres.13831.2

**Published:** 2024-12-05

**Authors:** Anthony Maher, Kevin Roche, Eimear C Morrissey, Andrew W Murphy, Greg Sheaf, Cristin Ryan, Gerry Molloy

**Affiliations:** 1 School of Psychology, University of Galway, Galway, County Galway, Ireland; 2 Centre for Health Research Methodology, School of Nursing and Midwifery, University of Galway, Galway, County Galway, Ireland; 3 Health Research Board Primary Care Clinical Trials Network Ireland, University of Galway, Galway, Ireland; 4 The Library of Trinity College Dublin, Dublin, Ireland; 5 School of Pharmacy and Pharmaceutical Sciences Panoz Institute, Trinity College Dublin, Dublin, Ireland

**Keywords:** Antimicrobial stewardship; anti-bacterial agents; behavioural sciences; intervention design; primary care.

## Abstract

**Objective:**

This scoping review aims to synthesise the extent and type of evidence on behaviour change interventions which address antibiotic treatment seeking behaviour for respiratory tract infections in primary care and/or community care settings.

**Introduction:**

Antimicrobial Resistance is recognised as a global health and economic threat by the World Health Organization and World Bank. Multiple factors, including patient and public demand, may contribute to unnecessary prescribing practices, which can lead to an overuse of antibiotics, and affect AMR. Current policy initiatives acknowledge the need to prepare for the future by managing public expectations regarding antibiotics, especially for influenza-like illness and other respiratory tract infections. These initiatives emphasise the importance of designing and evaluating effective interventions that generate actionable knowledge for policy and practices related to the appropriate use of antibiotics. Behaviour change interventions, in this context, can aim to modify patients' attitudes, beliefs, and behaviours regarding antibiotics.

**Inclusion criteria:**

Identified studies will describe behaviour change interventions aimed at potential patients and/or carers within the primary care and/or community care setting that address antibiotic treatment seeking behaviour for respiratory tract infections.

**Methods:**

This scoping review will search the literature in Medline, Embase, CINAHL, PsycINFO, Web of Science Core Collection, Scopus, EThOS, and Google Scholar to explore behaviour change interventions used to reduce expectations of antibiotics for respiratory tract infections in primary care. This review will follow the Joanna Briggs Institute guidelines for scoping reviews. It will be reported according to the Preferred Reporting Items for Systematic reviews and Meta-Analyses extension for Scoping Reviews.

## Introduction

The World Health Organization (WHO) has declared the unnecessary use of antimicrobials as a primary driver in the development of drug-resistant pathogens, accelerating the prevalence of antimicrobial resistance (AMR) ^1^. It is estimated that up to 1.3 million fatalities are due to AMR each year, with a projected tenfold increase by 2050 ^2, 3^. As an ongoing and escalating critical global health issue, multi-sectoral action is required in order to combat this global challenge ^4^. The creation of actionable knowledge which can enhance the appropriate use of antibiotics, may help curtail the spread of AMR ^1, 5^. Behaviour change interventions (BCIs), in this area, can be seen as one segment of a collective approach. Proactively, BCIs could promote prudent antibiotic use for respiratory tract infections (RTIs) amongst primary care networks and within community care settings ^5^.

Patient expectations can play a role in shaping antibiotic prescribing behaviours among healthcare professionals (HCPs) ^6, 7^. Studies in Australia and the UK indicate that many patients approach consultations with a strong belief that antibiotics are necessary for their symptoms ^8, 9^. An increased pressure on clinicians to meet these demands may result ^6, 9^. This could be referred to as antibiotic treatment-seeking behaviour (ATSB). This is a specification of one category of antibiotic demand whereby individuals pursue antibiotics for self-diagnosed conditions ^10^. This may be based on symptoms and regardless of clinical need. ATSB, similar to the broader concept of antibiotic demand, could result from a multitude of factors, including cultural norms, health beliefs, and/or prior prescription patterns ^8, 10^. Previous research illustrates that antibiotic demand is influenced by factors like symptom severity, prior healthcare experiences, and communication with healthcare staff ^8, 11, 12^. This complex interplay between patients, HCPs and clinical decision-making highlights the necessity for tailored interventions that educate patients, fostering more informed discussions about the appropriate use of antibiotics.

RTIs are among the most common reasons for antibiotic prescriptions in primary care settings ^13, 14^. Despite the majority of RTIs being viral in aetiology, antibiotics are frequently prescribed unnecessarily ^14^. Observational studies in the UK highlight high rates of unnecessary prescribing for RTIs, specifically in primary and community care ^15, 16^. Although patient expectations and providers perceptions of demand may be contributing factors, the UKs study data signifies the disparity that can arise between clinical guidelines and prescribing practices ^15^. This in turn may impact coding system reports, such as those offered by the ICD-10 criteria for RTIs ^17^. Taken together, these factors impact the categorisation of AMRs impact. Unnecessary antibiotic use may also expose patients to medication-related risks, such as adverse drug effects and increased healthcare costs ^18– 20^. Addressing antibiotic-associated costs and harms, may require multifaceted interventions, targeting both prescribers, patients, and healthcare system factors, particularly for RTIs. This is recognised by current international policy initiatives, as well as WHO AMR related surveillance reports ^21^. The results of inter-continental surveillance has also underscored the importance of effective behavioural science-based research in this area ^2, 21, 22^. Addressing these behavioural drivers may help to mitigate the impact of AMR.

The recognition of social and behavioural sciences' perspectives has led to the development of psychological interventions targeting AMR in recent years ^23, 24^. Such interventions may be considered as BCIs, or “co-ordinated sets of activities designed to change specified behaviour patterns” ^25^. For instance, the utilisation of delayed prescriptions can counteract 'action bias' – defined as “an impulse to act in order to gain a sense of control over a situation and eliminate a problem ^26, 27^. To enhance the understanding of BCIs, the Capability-Opportunity-Motivation Behaviour (COM-B) model can serve as index for mapping key determinants of health-seeking behaviours that these interventions may target ^25, 28^. Prior studies indicate that this model can guide the analysis of psychological interventions aimed at improving antibiotic prescribing among HCPs ^28, 29^. By providing a structured approach to the understanding of BCIs through the COM-B model, both prescriber and patient-targeted initiatives could align with clinical best practices.

Within the human healthcare setting, it has been highlighted that patient expectations for antibiotics can influence prescribing practices ^30^. Misalignments can occur between patients’ beliefs about the necessity of antibiotics and HCPs’ assessments ^31^. Patient expectations may not always be accurately perceived by prescribers, which may lead to an overestimation of the demand for antibiotics ^32, 33^. A clearer understanding of patient-driven antibiotic use could help develop more targeted, inter-disciplinary approaches to curb AMR. A recent systematic review of interventions reported on the improvement of antibiotic use patterns from behaviour change interventions (BCIs) within low- and middle-income countries, however these were not specifically addressing the context of RTIs ^34^. With a focus on addressing ATSB, a randomised experiment in the UK examined behaviour change approaches for reducing hypothetical expectations for antibiotics in primary care ^35^.

Given the spread of methodologies and conceptual diversity present within unnecessary antibiotic use, patient expectation, and antibiotic demand, a scoping review was identified as the most appropriate methodology to address this knowledge gap ^36, 37^. This will allow for an assessment of diverse interventions, identification of knowledge gaps, and evaluation of methodological approaches, particularly in an emerging area where the evidence appears to be fragmented. To our knowledge, there is no other systematic or scoping review that looks to synthesise the available evidence on BCIs within primary and/or community care settings, to reduce ATSB for RTIs.

## Review question

This review aims to synthesise the extent and type of evidence on BCIs which address ATSB for RTIs in primary and/or community care settings.

The research questions forming the objectives of the review are:

- What BCIs have been applied and/or evaluated to reduce ATSB for RTIs?
- What attributes of BCIs can be characterised by coding and mapping them to the COM-B model within the ‘Behaviour Change Wheel’ framework?
- What behaviour change theories are embedded within interventions that are used to reduce ATSB for RTIs, if any?
- What gaps exist presently in the literature that need to be addressed in future research for BCIs?

## Eligibility criteria

### Participants

Eligible studies will focus on the patient or carers within the primary and/or community care setting, that present with or discuss symptom(s) that typically constitute an RTI, as defined by ICD-10 RTI criteria ^17^.

### Concept

An objective or self-reported change of an individual or public groups ATSB relating to RTIs – this may be set in the form of improved (or intention to improve) necessary antibiotic use, reduced antibiotic consumption, increased knowledge of antibiotic use, or improved attitude/belief towards antibiotic or AMR. This excludes BCIs which target HCPs only or where the outcome of the intervention cannot be separated HCPs and patients or carers.

### Context

Relevant studies in the English language from any country that focus on primary and/or community care will be included.

### Types of sources

All study types including quantitative, qualitative, mixed methods, descriptive studies, and grey literature where they describe empirical work, will be included.

## Methods

The proposed scoping review will be conducted in accordance with the JBI methodology for scoping reviews ^38^. A preliminary search of MEDLINE, PROSPERO, the Cochrane Database of Systematic Reviews and JBI Evidence Synthesis was conducted and no current or underway systematic reviews or scoping reviews on the topic were identified. This scoping review protocol was registered on the Open Science Framework and is available at https://doi.org/10.17605/OSF.IO/DZF9C.

### Search strategy

The search strategy will be developed in collaboration with a research librarian. An initial limited search of MEDLINE and CINAHL will be undertaken to identify articles on the topic. The text words contained in the titles and abstracts of relevant articles, and the index terms used to describe the articles will be used to develop a full search strategy for Medline, Embase, CINAHL, PsycINFO, Web of Science Core Collection, Scopus, EtHOS, and Google Scholar (see Appendix I). The search strategy, including all identified keywords and index terms, will be adapted for each included database and/or information source. The reference list of all included sources of evidence will be screened for additional studies.

Studies published in the English language will be searched for and included. Only studies published since 2000 will be included for pragmatic reasons.

### Study/Source of evidence selection

Following the search, all identified citations will be collated and uploaded into Covidence®, with associated duplicates removed. Titles and abstracts will then be screened by two or more independent reviewers for assessment against the inclusion criteria for the review. Potentially relevant sources will be retrieved in full, and their citation details imported into the Covidence®. A minimum of 10% of full texts from the selected studies will be evaluated against the inclusion criteria by two or more independent reviewers. The primary author will review 100% of the full text articles. Reasons for exclusion of sources of evidence at full text that do not meet the inclusion criteria will be recorded and reported. Any disagreements that arise between the reviewers at each stage of the selection process will be resolved through discussion, or with an additional reviewer/s input. The results of the search and the study inclusion process will be reported in full and presented according to the Preferred Reporting Items for Systematic Reviews and Meta-analyses extension for scoping review (PRISMA-ScR) flow diagram ^39^.

### Data extraction

A minimum of 10% of full texts will undergo data extraction from two or more independent reviewers, using a data extraction tool. The primary author will complete 100% extraction of all selected full texts. A data extraction tool will be developed by the reviewers, with inter-rater reliability results tabulated and reported. The data extracted will include specific details about the participants, concept, context, study methods and key findings relevant to the review question/s.

A draft extraction form is provided (see Table 1). The draft data extraction tool will be modified and revised as necessary during the process of extracting data from each included evidence source. Modifications will be detailed in the results paper. Any disagreements that arise between the reviewers will be resolved through discussion, or with an additional reviewer/s. If appropriate, authors of papers will be contacted to request missing or additional data, where required.

**Table 1.** Sample tool to be used for data extraction.

Title	Study Details (Author, year)	Location	Sample size	Study design	Intervention description	Behavioural target	COM-B Framework components	Intervention function	Mode of Delivery	Theoretical basis	Outcome
											
											
											
											

### Data analysis and presentation

The mapped data will be analysed to identify patterns, trends, and relationships between the characteristics of BCIs and the domains of the COM-B-model. A clear distinction will be made between theory-driven interventions, which are based on established behavioural models, and pragmatically developed interventions, which may lack a mentioned theoretical grounding. Common themes and concepts will be synthesised to provide a comprehensive understanding of how the interventions align with the COM-B model, and where the evidence spreads to.

The presentation of the scoping review will include a synthesis in the form of a narrative summary. This will accompany the tabulated and/or charted results and will describe how the results relate to the reviews objective and question/s. of the findings, including key themes, trends, and recommendations for future research and practice.

## Data Availability

No data are associated with this article.

## Appendices

### Appendix I: Draft Search strategy

(((Respiratory OR chest) NEAR/2 (disease* OR infection*)) OR "RTI*" OR Bronchitis OR "Common cold*" OR Croup OR Epiglottitis OR Flu OR Influenza* OR Laryngitis OR Laryngotracheitis OR Otalgia OR Pharyngitis OR Pleurisy OR Pneumo* OR Rhinitis OR Sinusitis OR Tonsillitis OR Breathless* OR Congestion OR Cough* OR Earache* OR Headache* OR "High temperature*" OR "Muscle ache*" OR "Runny nose*" OR Sneez* OR "Sore throat*" OR Wheez*)

AND

(((Family OR General) NEAR/2 (doctor* OR physician* OR pract*)) OR ((Community OR Primary) NEAR/2 (care OR center* OR centre* OR healthcare)) OR Ambulatory OR Pharmac* OR (Home NEAR/2 visit*))

AND

((antibiotic* OR "anti biotic*" OR antimicrob* OR "anti microb*") NEAR/5 (resistan* OR prescrib*))

AND

(accept* OR access* OR approach* OR availab* OR behav* OR chang* OR communicat* OR convenien* OR decision* OR engag* OR guid* OR influen* OR intention* OR interven* OR motiv* OR nudg* OR persua* OR plan* OR polic* OR procedure* OR program* OR strateg* OR support* OR train* OR uptake*)

MEDLINE (PubMed): MeSH terms

"Respiratory Tract Infections", "Bronchitis", "Influenza, Human", "Pneumonia", "Sinusitis", "Pharyngitis", "Tonsillitis", "Laryngitis", "Rhinitis", "Otitis Media", "Croup", "Physicians, Family", "General Practitioners", "Primary Health Care", "Community Health Services", "Ambulatory Care", "Pharmacies", "Anti-Bacterial Agents", "Drug Resistance, Bacterial", "Drug Prescriptions", "Drug Utilization", "Health Behavior", "Behavioral Medicine", "Patient Participation", "Patient Education as Topic", "Health Communication", "Decision Making"

EMBASE: Emtree terms

"respiratory tract infection", "bronchitis", "influenza", "pneumonia", "sinusitis", "pharyngitis", "tonsillitis", "laryngitis", "rhinitis", "otitis media", "croup", "general practitioner", "family physician", "primary health care", "community care", "ambulatory care", "pharmacy", "antibiotic agent", "bacterial resistance", "drug prescription", "drug utilization", "health behavior", "behavioral medicine", "patient participation", "patient education", "health communication", "decision making".

CINAHL (via EBSCOhost): CINAHL headings

"Respiratory Tract Infections", "Bronchitis", "Influenza", "Pneumonia", "Sinusitis", "Pharyngitis", "Tonsillitis", "Laryngitis", "Rhinitis", "Otitis Media", "Croup", "Family Practice", "General Practice", "Primary Health Care", "Community Health Services", "Ambulatory Care", "Pharmacy Services", "Antibiotic Agents", "Drug Resistance", "Drug Prescribing", "Health Behavior", "Patient Participation", "Patient Education", "Health Communication", "Decision Making"

PsycINFO: APA Thesaurus terms

"Antibiotic Resistance", "Antibiotic Prescribing", "Behavior Change", "Behavioral Intentions", "Clinical Decision Making", "Communication", "Community Health", "Cough", "Family Medicine", "Health Behavior", "Health Education", "Influenza", "Patient Attitudes", "Patient Education", "Primary Care", "Respiratory Tract Infections", "Sinusitis", "Tonsillitis", "Treatment Adherence"

Web of Science Core Collection: keywords and Boolean operators

"Antibiotic Resistance", "Antibiotic Prescribing", "Behavior Change", "Behavioral Intent", "Clinical Decision Making", "Communication", "Community Health", "Cough", "Family Medicine", "Health Behavior", "Health Education", "Influenza", "Patient Attitudes", "Patient Education", "Primary Care", "Respiratory Tract Infections", "Sinusitis", "Tonsillitis", "Treatment Adherence"

Scopus: Scopus’s advanced search options for terms combined with Boolean operators

"Antibiotic Resistance", "Antibiotic Prescribing", "Behavior Change", "Behavioral Intention", "Clinical Decision Making", "Communication", "Community Health", "Cough", "Family Medicine", "Health Behavior", "Health Education", "Influenza", "Patient Attitudes", "Patient Education", "Primary Care", "Respiratory Tract Infections", "Sinusitis", "Tonsillitis", "Treatment Adherence"

EThOS: simple phrases, with quotation marks around exact terms.

Google Scholar: simple phrases, with quotation marks around exact terms.
