# Supplementary material for: Behaviour change interventions addressing antibiotic treatment seeking behaviour for respiratory tract infections in primary care settings: A scoping review protocol
Source: HRB Open Res. 2024 Dec 5;7:41. Originally published 2024 Jul 1. [Version 2] doi: 10.12688/hrbopenres.13831.2 (PMC11869197; doi:10.12688/hrbopenres.13831.2)
Supplement: Peer review history

# Reviewer report: Reviewer response for version 2

**DOI:** 10.21956/hrbopenres.15413.r44715

**Published:** 2025-02-26

**Authors:** Emily K. Rousham

**Affiliations:** 1 Loughborough University, Loughborough, UK

Thank you for the opportunity to review this work. This is nicely presented protocol for a scoping review on a topic very relevant to the problem of antimicrobial resistance and appropriate antibiotic use. The protocol follows appropriate guidelines and methodologies (JBI and PRISMA ScR). The rationale and methods are clearly described but some minor clarifications could strengthen the protocol further. The  concept could be clarified in terms of the wording as 'an objective or self-reported change of an individual or group antibiotic-seeking treatment behaviour' - should this include '...resulting from an intervention'? Also, the phrasing of the concept suggests that studies will only be included if a change in behaviour is observed or reported, which would bias results. Will the outcomes of all interventions be reported, whether they result in a change in behaviour or not? (i.e. null findings will also be included).

 Finally, whilst acknowledging that the data extraction form is still in draft form, Table 1 could be modified to add some data extraction relating to participant/patient characteristics that may influence the outcome of an intervention e.g. age; patients vs parents/carers etc.

Is the study design appropriate for the research question?

Yes

Is the rationale for, and objectives of, the study clearly described?

Partly

Are sufficient details of the methods provided to allow replication by others?

Partly

Are the datasets clearly presented in a useable and accessible format?

Not applicable

Reviewer Expertise:

Antimicrobial resistance, interventions, systematic and scoping reviews.

I confirm that I have read this submission and believe that I have an appropriate level of expertise to confirm that it is of an acceptable scientific standard, however I have significant reservations, as outlined above.

# Reviewer report: Reviewer response for version 2

**DOI:** 10.21956/hrbopenres.15413.r44344

**Published:** 2025-01-10

**Authors:** Isabel Naomi Aika

**Affiliations:** 1 University of Benin, Benin City, Edo, Nigeria

I have read through the research protocol on "Behaviour change interventions addressing antibiotic treatment seeking behaviour for respiratory tract infections in primary care settings: A scoping review".

 I believe this will be an extensive research of application of BCI to preserve antibiotic use, and its focus on patients sounds interesting.

 The study protocol write-up is well thought out, with clearly defined terms of the research framework.

 However the authors can consider the following observations:

 The use of RTI is broad. is the study focus on RTIs generally, or will it include upper respiratory tract infections and lower respiratory tract infections? I see in the search strategies, terms for specific respiratory tract infections were included. I was wondering that studies without those terms but specifically addressing either URTIs and/or LRTIs might be omitted. These could be added to the search terms.

 Secondly, since the study is adopting social and behavioural change, could this also include behavioural change communication?

 In the analysis section, no mention of assessing risk of bias of studies that will be included in your review?

Is the study design appropriate for the research question?

Yes

Is the rationale for, and objectives of, the study clearly described?

Yes

Are sufficient details of the methods provided to allow replication by others?

Yes

Are the datasets clearly presented in a useable and accessible format?

Yes

Reviewer Expertise:

health services, pharmacy practice, antimicrobial stewarship,

I confirm that I have read this submission and believe that I have an appropriate level of expertise to confirm that it is of an acceptable scientific standard, however I have significant reservations, as outlined above.

# Reviewer report: Reviewer response for version 2

**DOI:** 10.21956/hrbopenres.15413.r43511

**Published:** 2024-12-14

**Authors:** Regina Poß-Doering

**Affiliations:** 1 Department of General Practice and Health Services Research, University Hospital Heidelberg, Heidelberg, Germany

Reviewer comments have been addressed. I have no further comments at this point.

Is the study design appropriate for the research question?

Yes

Is the rationale for, and objectives of, the study clearly described?

Yes

Are sufficient details of the methods provided to allow replication by others?

Yes

Are the datasets clearly presented in a useable and accessible format?

Not applicable

Reviewer Expertise:

health services research; implementation science; process evaluations; rational use of antibiotics; holistic chronic non-cancer pain case and care management (in primary care)

I confirm that I have read this submission and believe that I have an appropriate level of expertise to confirm that it is of an acceptable scientific standard.

# Reviewer report: Reviewer response for version 1

**DOI:** 10.21956/hrbopenres.15151.r42026

**Published:** 2024-09-10

**Authors:** Aleksandra Borek, Caity Roleston

**Affiliations:** 1 University of Oxford, Oxford, UK

**Your Report**

 Overall, the proposed review addresses an important question and would likely be a valuable contribution to the research literature. The scoping review is an appropriate approach to address the stated objective of synthesising the extent and type of evidence. However, in its current form, the proposal has major flaws: the rationale for the focus and scope of this review is unclear; the objectives are unclear and inconsistent; the study design and methods are not described in a sufficient detail and clarity to allow replication. The issues we have identified with the literature cited (e.g., mis-cited, lack of key relevant references) cast further doubts on the quality of this protocol. Therefore, we recommend that, at the minimum, the Authors revise the protocol to ensure that: (1) the references cited are correct, (2) the methodological approach and reporting guidelines are clearly stated and followed, and (3) the methods are sufficiently and transparently reported.

 Below, we list our specific comments and questions related to each section of the protocol to help improve this protocol. 

**Abstract**

 1.  ‘Demand as a key driver of inappropriate antibiotic use’ – It is widely recognised that antibiotic prescribing/use is influenced by a complex combination of multiple factors. Therefore, stating that demand is a key driver is reductionist and incorrect. We suggest rephrasing this to, e.g.: patient expectations and demand for antibiotics are among important drivers…

**Introduction**

 2. The Introduction should include a clear explanation of the rationale for conducting a scoping review – why this approach (as opposed to, e.g., a systematic review) was chosen and is appropriate. This is missing yet suggested in the best practice and reporting guideline for scoping review protocols by Peters et al. 2022 and in PRISMA-ScR checklist by Tricco et al. 2018.

 3. There is a lack of a clear rationale for focusing on patient treatment seeking and expectations/demand for antibiotics. For example, what is known and not known about the role of patient expectations and demand in affecting antibiotic prescribing? Patient expectations may be over-estimated by prescribers, especially when prescribers do not ask about them directly, which should be also acknowledged. Otherwise, it is reductionist to point unequivocally to patient demand as a key driver (as per comment #1).

 4. Second paragraph – it is unclear how it relates to the main focus of the review. How exactly is the AWaRe classification relevant? It could be replaced with more discussion of the patient-related drivers of antibiotic prescribing/use.

 5. The key terms used should be defined/clarified:

- What do the Authors mean by ‘inappropriate’ antibiotic prescribing? E.g., inappropriate compared to guidelines? If not compared to guidelines, perhaps ‘unnecessary’ or ‘with little benefit’ might be better than ‘inappropriately’ prescribed. 
- The key concepts – patient expectations, demand and treatment seeking behaviours – should be defined in the Introduction, possibly together with providing a rationale for focusing on these concepts (as per comment #2). For example, treatment seeking behaviour seems to be used in a broad sense, incorporating psychological determinants of behaviours (such as patient beliefs, expectations), not only behaviour.
- What do ‘supply side’ solutions mean?
- It would be useful to define BCIs, especially as they are key to this review. The Authors should clarify (if this is correct) that by BCIs they also consider interventions that target behavioural determinants (e.g., beliefs, expectations). Modifying such determinants may or may not affect actual behaviours. It would be also good to define what types of patient behaviours the interventions would need to target (e.g., consulting behaviour?) as changing expectations alone is not behaviour change. Or consider keeping the description broader to include ‘interventions’ (not specifically BCIs) that target patients and their beliefs and behaviours related to seeking help and treatment for RTIs.

  6. The are several issues with cited literature, including citing literature that seems tangentially connected, mis-citing studies, and missing relevant references. For example:

- The sentence ’It is estimated…’ needs a reference. We also recommend that the authors ensure that ‘due to AMR’ is correct (and that it should not be ‘associated with AMR’).
- The claim that antibiotic treatment seeking behaviour has been identified as a key area for investigation is not well supported by the references cited – Bell et al (2014) (reference 4) does not seem to support this claim (the authors emphasise the importance of responsible prescribing at individual and national levels). The other reference is the World Bank which wrote ‘consumers have to stop demanding antibiotics when they have a viral infection…’ – it is not immediately clear what evidence these claims are based on – see also our other comments regarding the issue of ‘demand’ for antibiotics.
- ‘RTIs are known to represent one of the most common reasons…’ – a reference is missing. There is evidence to support such a claim; e.g., in England, the ESPAUR reports show every year that most antibiotics are prescribed in primary care/ in the community; similarly, observational studies [ ref 1 ] show that.
- ‘Up to 60% of cases’ – could you provide a range or an approximate number rather than ‘up to’ figure only (‘up to’ could be any number under 60% so it’s not precise or informative). Also, we question whether the references to support this claim/sentence are the most relevant and appropriate. For example, there are large observational studies that specifically assessed the proportions of antibiotics being prescribed not according to guidelines that constitute better evidence for such a claim (e.g., a special issue on this topic - [ ref 2 ] ).
- ‘Managing public expectations for the prescribing of antibiotics (…) recognised by current international policy initiatives, as well as WHO AMR related surveillance reports’ – the reference is neither current international policy nor WHO AMR report, but rather an article about how behavioural sciences can help with antimicrobial prescribing practice. Although it might be relevant, if the authors refer to policy initiatives and WHO in this sentence, then they should reference these sources.  
- ‘The recognition of social and behavioural sciences’ perspectives has led to the development, evaluation, and implementation of effective psychological interventions aimed at reducing antibiotic prescribing within the context of AMR management’ – references cited do not seem to support this claim. Costelloe (ref. 16) is not on the effectiveness of behavioural interventions. Hullscher (ref. 17) relates to hospitals, not primary care/community (which is the focus of this review). Atkins (ref. 18) introduces the Theoretical Domains Framework to investigate implementation problems and does not report an effective psychological intervention using behavioural sciences to reduce AMR. There are several relevant papers on this topic that could be referred to, e.g., [ref 3] , [ref 4] , [ref 5], [ref 6]
-  ‘This framework (COM-B) has provided a theoretical lens for examining behaviour change interventions in primary care settings aimed at addressing antibiotic prescribing behaviours among providers’ – the references cited do not seem to have used COM-B, certainly not explicitly. If the Authors are saying that we can better understand efficacious interventions by mapping the successful component(s) to COM-B, then that needs to be explained more clearly here. The way it is currently written implies the cited studies involved developing efficacious interventions using COM-B to address antibiotic prescribing.
- ‘a randomised controlled trial (RCT) in the UK examined behaviour change approaches for reducing expectations for antibiotics in primary care’ (ref. 28) – This description of the study is misleading. First, it was a randomised experiment, not a trial/RCT. Second, it involved hypothetical scenarios (different types of messages and their impact on intentions) and not actual behaviour.

**Review question and objectives**

 7. ****The review aim and objectives in this section should be clarified; specifically:

- The aim in this section differs from the one provided in the Abstract. The one provided in the abstract seems more suited to the choice of a scoping review.
- Treatment seeking behaviour is mentioned in the abstract, whereas expectations for antibiotics are mentioned in the main text – these are different things. Do the Authors mean that they aim to identify BCIs that seek to lower patient/carer expectations for antibiotic prescription for RTI symptoms? The key concepts and focus of the review should be introduced in the Introduction (as per comment 5b) and then kept consistent in the Review aims and objectives.
- Do the Authors aim to map the intervention components to the COM-B model? What is the purpose of this and how is this objective suited to the choice of a scoping review (rather than a systematic review)?

8. ‘As diagnosed by an ICD-10 criterion’ – Can the Authors explain what the ICD-10 criterion is? Is the review aim a relevant place to include this in? (It seems to be something that might be better explained in the Introduction.) It is also unclear what this phrase is meant to indicate – it is needed at all? Patient expectations for antibiotics are likely to be influenced by their (RTI) symptoms, not a diagnosis.

**Eligibility criteria**

 9. ‘Conceptually present with an RTI’ – It is unclear what ‘conceptually present’ means. Perhaps it would be clearer to state instead ‘present with RTI symptoms’.  

 10. ‘As diagnosed by an ICD-10 criterion’ – How is the ICD-10 operationalised here? What do the Authors mean by ‘as diagnosed’? Is ‘as diagnosed’ an appropriate phrase here? (It suggests that ICD-10 diagnoses an RTI.)  

 11. Concept – Are consultation and help/advice seeking behaviours included here? E.g. whether patients seek consultations for RTI symptoms, which may or may not involve/imply seeking, expecting or demanding antibiotics. (This links with our previous concepts around clarifying the concepts and focus of this review).

**Search strategy**

 12. Inclusion of all languages – How will research in any language be read and data extracted? Does the review team have sufficient resources to do so?  

 13. ‘Given the timing at which the implementation of the theoretical domains framework took place across interventions’ – What do the Authors mean by this? Interventions might have been developed and tested regardless of the theoretical frameworks. In fact, interventions are often developed without the use of theory or frameworks. Moreover, which theoretical frameworks do the Authors refer to? There are many health behaviour change theories developed before 2000, and the Behaviour Change Wheel was published in 2014.  

 14. Linked with the previous comment, the rationale provided for applying a time limit is unconvincing. While we understand that time limits might be necessary, the reason for that should be clear (even if it is related to, e.g., feasibility, relevance of the expected literature etc.).

**Methods**

 15. ‘The proposed scoping review will be conducted in accordance with the JBI methodology for scoping reviews’ – The reference provided for this is incorrect. The authors cite “The development of software to support multiple systematic review types: the Joanna Briggs Institute System for the Unified Management, Assessment and Review of Information (JBI SUMARI)” which is an article detailing the development of a software program to support systematic reviews not the JBI guidance/manual for conducting scoping review.  

 16. ‘JBI System for unified management…’ – Reference for this is incorrect – currently number 25 which relates to ‘Antimicrobial resistance: risk associated with antibiotic overuse and initiatives to reduce the problem’ – presumably the Authors intended to cite the reference miscited above (point 15) pertaining to JBI SUMARI.  

 17. In line with the most recent recommendations for scoping review methodology (see Peters et al. (2022) ‘Best practice guidelines and reporting items for the development of scoping review protocols’), we suggest the Authors begin the methods section by clearly identifying and correctly referencing the scoping review methodology/framework they have used (e.g., JBI, Arksey & O’Malley, Levac et al), and then throughout the protocol, the methodology/framework should be followed and sufficiently reported.

**Study/source of evidence selection**

 18. Why do the Authors propose to use both Covidence and JBI SUMARI if both systems can be used for all steps of the review process?  

 19. ‘Titles and abstracts will be screened by two or more independent reviewers’ – Does this mean that each title/abstract will be screened by at least two (possibly more) reviewers (i.e. duplicate screening)?

**Data extraction**

 20. Will the data extraction be conducted in duplicate? Other than the ‘10% of included papers’, will the remaining data extraction be done by a single reviewer?  

 21. Table 1:

- Population – suggest reporting also sample sizes;
- Concept – type of BCI – how will it be determined? What is meant by ‘to inform theoretical framework’?

**Data analysis**

 22. BCIs could be coded using the full Behaviour Change Wheel model, not only COM-B; e.g., intervention functions are useful and relevant. (However, it remains unclear to us how exactly this fits with the aim of the scoping review stated in the Abstract of synthesising the extent and type of evidence – as per comments 7a and 7c).  

 23. How exactly will the COM-B model (concepts) be used in the analysis of the interventions? Will the intervention descriptions be coded using these theoretical concepts? We would suggest that a codebook and coding instructions are developed, with definitions of concepts (e.g. COM-B, intervention functions – for example from the Behaviour Change Wheel book) to ensure a correct and reliable coding process.

**Minor points:**

- Introduction – it should be ‘an increasing trend of sporadic…’
- ‘Behaviour change in this domain’ – ‘domain’ is an odd word choice; perhaps a more straightforward word (e.g. ‘area’) would be clearer
- ‘Compared to the preceding two years, namely 2020 and 2019’ – the sentence establishes this as ‘2022 data’ so by stating the comparison is with the preceding two years means the inclusion of ‘namely 2020 and 2019’ is redundant.
- Abbreviation RTIs should be used consistently after being introduced, e.g. ‘up to 60% of cases of acute respiratory infections.’
- The last sentence of the Introduction: a comma in ‘aimed, to’ should be removed; suggest revising the sentence as it repeats ‘aimed’ and is quite long and complex.
- The sub-heading is ‘review question’ but the section does not provide questions – it reports the aim and objectives.
- Review questions - Why is this section (and some subsequent sections) in italics?
- Eligibility criteria: should be ‘focus on the patients or carers’, not patient.
- Context – ‘focuses’ should be ‘focus’ as refers to publications; no comma needed.
- ‘One independent reviewer will complete remaining screening’ – should be just ‘one reviewer’; being ‘independent’ only is relevant if multiple people double screen the same records.

Is the study design appropriate for the research question?

Yes

Is the rationale for, and objectives of, the study clearly described?

No

Are sufficient details of the methods provided to allow replication by others?

No

Are the datasets clearly presented in a useable and accessible format?

Not applicable

Reviewer Expertise:

NA

We confirm that we have read this submission and believe that we have an appropriate level of expertise to state that we do not consider it to be of an acceptable scientific standard, for reasons outlined above.

## References

[1] Reference source

[2] Reference source

[3] : The role of behavior change in antimicrobial stewardship. *Infect Dis Clin North Am* .2014;28(2) : 10.1016/j.idc.2014.01.004 169-75 10.1016/j.idc.2014.01.004 24857386

[4] : Contribution of behavioural science to antibiotic stewardship. *BMJ* .2015;350(jun25 8) : 10.1136/bmj.h3413 10.1136/bmj.h3413 26111947

[5] : How can behavioural science contribute to qualitative research on antimicrobial stewardship in primary care?. *JAC Antimicrob Resist* .2022;4(1) : 10.1093/jacamr/dlac007 dlac007 10.1093/jacamr/dlac007 35156031 PMC8826758

[6] : We must harness the power of social and behavioural science against the growing pandemic of antimicrobial resistance. *Nat Hum Behav* .2024;8(1) : 10.1038/s41562-023-01762-y 11-13 10.1038/s41562-023-01762-y 37985918

# Response

**Published:** 2024-12-09

**Authors:** Anthony Maher

**Affiliations:** School of Psychology, University of Galway, Galway, County Galway, Ireland

Many thanks for your insightful comments and for taking the time to review our protocol. We have updated the manuscript to reflect the changes that you have recommended. We have listed these changes below:

**Abstract**

**1. ‘Demand as a key driver of inappropriate antibiotic use’ – It is widely recognised that antibiotic prescribing/use is influenced by a complex combination of multiple factors. Therefore, stating that demand is a key driver is reductionist and incorrect. We suggest rephrasing this to, e.g.: patient expectations and demand for antibiotics are among important drivers…**

 Thank you for this helpful suggestion. On reflection, we acknowledge that this does not communicate the complexity of the factors that potentially influence antibiotic use. We have now modified the original sentence within the ‘abstract’ section, as follows: “Multiple factors, including patient expectations and public demand, may contribute to unnecessary prescribing practices, which can lead to an overuse of antibiotics, and affect AMR”

**Introduction**

**2. The Introduction should include a clear explanation of the rationale for conducting a scoping review – why this approach (as opposed to, e.g., a systematic review) was chosen and is appropriate. This is missing yet suggested in the best practice and reporting guideline for scoping review protocols by Peters et al. 2022 and in PRISMA-ScR checklist by Tricco et al. 2018**

 Thank you for highlighting this point. Based on the recommendations provided by Munn *et al*., 2018 and Munn *et al*., 2020, we have added the rationale for conducting a scoping review, as opposed to another form of literature synthesis within the introduction of this manuscript. This is located underneath the ‘introduction’ section as follows: “Given the spread of methodologies and conceptual diversity present within unnecessary antibiotic use, patient expectation, and antibiotic demand, a scoping review was identified as the most appropriate methodology to address this knowledge gap.”

**3. There is a lack of a clear rationale for focusing on patient treatment seeking and expectations/demand for antibiotics. For example, what is known and not known about the role of patient expectations and demand in affecting antibiotic prescribing? Patient expectations may be over-estimated by prescribers, especially when prescribers do not ask about them directly, which should be also acknowledged. Otherwise, it is reductionist to point unequivocally to patient demand as a key driver (as per comment #1).**

 We thank the reviewer for this point. The rationale for focusing on patients' health-seeking behaviours within the domain of antibiotic overprescribing has now been expanded within the introduction of the manuscript to enhance clarity. Points around the expectation mismatch between healthcare providers and patients have also been included to further enhance the clarity of the rationale, and help align the introduction with the title and abstract context. This is located underneath the ‘introduction’ section as follows: “ATSB, similar to the broader concept of antibiotic demand, could result from a multitude of factors, including cultural norms, health beliefs, and/or prior prescription patterns. Previous research illustrates that antibiotic demand is influenced by factors like symptom severity, prior healthcare experiences, and communication with healthcare staff.”

**4. Second paragraph – it is unclear how it relates to the main focus of the review. How exactly is the AWaRe classification relevant? It could be replaced with more discussion of the patient-related drivers of antibiotic prescribing/use.**

 We thank the reviewer for this point. For some clinical members of the review team, the AWaRe classification of medicines is seen as a common and standardised tool to clinically compare types of antibiotics, especially when it comes to the risk that it may pose to an individual in contracting adverse drug reactions, and contributing overall to AMR. On reflection, we have now chosen to omit this point and have expanded on the discussion of patient-related drivers of antibiotic prescribing and use further in this paragraph. This is located underneath the ‘introduction’ section as follows “Patient expectations can play a role in shaping antibiotic prescribing behaviours among healthcare professionals (HCPs). Studies in Australia and the UK indicate that many patients approach consultations with a strong belief that antibiotics are necessary for their symptoms. An increased pressure on clinicians to meet these demands may result”

**5. The key terms used should be defined/clarified:**

**5a) What do the Authors mean by 'inappropriate' antibiotic prescribing? E.g., inappropriate compared to guidelines? If not compared to guidelines, perhaps 'unnecessary' or 'with little benefit' might be better than 'inappropriately' prescribed.**

 We thank the reviewer highlighting this point. We have now modified “inappropriate” to “unnecessary” throughout the protocol manuscript, as evident in the third paragraph under the ‘introduction’ section, as follows: “Observational studies in the UK highlight high rates of unnecessary prescribing for RTIs, specifically in primary and community care.”

**5b) The key concepts - patient expectations, demand and treatment seeking behaviours - should be defined in the Introduction, possibly together with providing a rationale for focusing on these concepts (as per comment #2). For example, treatment seeking behaviour seems to be used in a broad sense, incorporating psychological determinants of behaviours (such as patient beliefs, expectations), not only behaviour.**

 We thank the reviewer for this recommendation. We have now modified the manuscript to reflect accompanying definitions with key concepts including antibiotic treatment seeking behaviour, to encompass the differentiation around antibiotic expectation and patient demand. This is located underneath the ‘introduction’ section, as follows: “This could be referred to as antibiotic treatment seeking behaviour (ATSB). This is a specification of one category of antibiotic demand whereby individuals pursue antibiotics for self-diagnosed conditions.”

**5c) What do 'supply side' solutions mean?**

 We thank the reviewer for highlighting the ambiguity of this sentence. Supply-side solutions refer to strategies aimed at influencing the healthcare providers (i.e., the prescribers), healthcare systems, and pharmaceutical suppliers to reduce unnecessary antibiotic prescriptions. This may include such things as prescriber education and training, decision support tools and audit and feedback mechanisms. On reflection, we have omitted “’supply side’ solutions” from the manuscript in our re-design of the introduction as this might introduce an over-simplistic false dichotomy. A similar meaning to our original point may now been seen in the third paragraph under the ‘introduction’ session, as follows: “Addressing antibiotic-associated cost and harms, may require multifaceted interventions, targeting prescribers, patients and healthcare system factors, particularly for RTIs.”

**5d) It would be useful to define BCIs, especially as they are key to this review. The Authors should clarify (if this is correct) that by BCIs they also consider interventions that target behavioural determinants (e.g., beliefs, expectations). Modifying such determinants may or may not affect actual behaviours. It would be also good to define what types of patient behaviours the interventions would need to target (e.g., consulting behaviour?) as changing expectations alone is not behaviour change. Or consider keeping the description broader to include 'interventions' (not specifically BCIs) that target patients and their beliefs and behaviours related to seeking help and treatment for RTIs.**

 We thank the reviewer for this suggestion. We acknowledge that modifying behavioural determinants may not change actual behaviour. However, in order to identify an inclusive set of cohesive intervention strategies, we aim to include studies that meet the definition of a BCI by Michie et al., 2011, but also to include studies that have behavioural determinants as an outcome e.g. patient expectations to receive an antibiotic. This is consistent with the broader focus that would typically characterise a scoping review, as the references below highlight.

 Greene C, Wilson J. The use of behaviour change theory for infection prevention and control practices in healthcare settings: a scoping review. Journal of infection prevention. 2022 May;23(3):108-17.

 Tzeng HM, Okpalauwaekwe U, Lyons EJ. Barriers and facilitators to older adults participating in fall-prevention strategies after transitioning home from acute hospitalization: a scoping review. Clinical interventions in aging. 2020 Jun 25:971-89.

 Roos M. A scoping review using the COM-B model to explore the literature on barriers and facilitators of help-seeking behavior amongst women affected by domestic and family violence in Australia

 Zinsser LA, Stoll K, Wieber F, Pehlke-Milde J, Gross MM. Changing behaviour in pregnant women: A scoping review. Midwifery. 2020 Jun 1;85:102680.

 Paton J, Abey S, Hendy P, Williams J, Collings R, Callaghan L. Behaviour change approaches for individuals with diabetes to improve foot self-management: a scoping review. Journal of Foot and Ankle Research. 2021 Dec;14:1-4.

**6) The are several issues with cited literature, including citing literature that seems tangentially connected, mis-citing studies, and missing relevant references.**

**6a) For example: The sentence 'It is estimated‚Ä¶' needs a reference. We also recommend that the authors ensure that 'due to AMR' is correct (and that it should not be 'associated with AMR').**

 We thank the reviewer for highlighting this issue within the manuscript. We have now amended the sentence “It is estimated..” to include what we feel is an appropriate, relevant reference - O’Neill *et al*., 2016 and Murray *et al*., 2022. We have also amended the wording of the sentence to be “associated with AMR”, as is evident under the ‘introduction’ section. References throughout the protocol’s manuscript have now been updated to enhance the reliability of statements made.

**6b) The claim that antibiotic treatment seeking behaviour has been identified as a key area for investigation is not well supported by the references cited - Bell et al (2014) (reference 4) does not seem to support this claim (the authors emphasise the importance of responsible prescribing at individual and national levels). The other reference is the World Bank which wrote 'consumers have to stop demanding antibiotics when they have a viral infection‚' - it is not immediately clear what evidence these claims are based on - see also our other comments regarding the issue of 'demand' for antibiotics.**

 We thank the reviewer for highlighting this point. Further evidence has now been identified to support the claim that antibiotic treatment seeking behaviour is identified as a key area for investigation. The manuscript has now been amended to reflect this, as evident underneath the ‘introduction’ section in the second and fifth paragraph.

**6c) 'RTIs are known to represent one of the most common reasons‚' - a reference is missing. There is evidence to support such a claim; e.g., in England, the ESPAUR reports show every year that most antibiotics are prescribed in primary care/ in the community; similarly, observational studies [ ref 1 ] show that.**

 We thank the reviewer for highlighting this point. On reflection, we have chosen to cite references to reflect the claim that “RTIs are among the most common reasons for antibiotic prescriptions in primary and community care settings”. The first reference is an observational cross-sectional survey that examined general practices in the UK, analysing the rates of antibiotic prescriptions for respiratory tract infection consultations. The second is a European Centre for Disease Prevention and Control report from 2021 that highlights the percentage of antibiotics attributable to RTIs versus other common indications e.g. urinary tract infections. This is evident underneath the ‘introduction’ section, as follows: “RTIs are among the most common reasons for antibiotic prescriptions in healthcare settings.(13,14)”

**6d) 'Up to 60% of cases' - could you provide a range or an approximate number rather than 'up to' figure only ('up to' could be any number under 60% so it's not precise or informative). Also, we question whether the references to support this claim/sentence are the most relevant and appropriate. For example, there are large observational studies that specifically assessed the proportions of antibiotics being prescribed not according to guidelines that constitute better evidence for such a claim (e.g., a special issue on this topic - [ ref 2 ] ).**

 We thank the reviewer for this comment. This particular part of the paragraph has been re-worked to reflect the current landscape of antibiotic use in the treatment of RTIs in our re-design of the manuscript. References to cases are no longer within the introduction section or elsewhere on the manuscript.

**6e) 'Managing public expectations for the prescribing of antibiotics (‚Ä¶) recognised by current international policy initiatives, as well as WHO AMR related surveillance reports' - the reference is neither current international policy nor WHO AMR report, but rather an article about how behavioural sciences can help with antimicrobial prescribing practice. Although it might be relevant, if the authors refer to policy initiatives and WHO in this sentence, then they should reference these sources.**

 We thank the reviewer for this comment. This particular part of the paragraph has been re-worked, with two references that support this sentence. The first is WHO global report on AMR, specifically revolving on its surveillance from a 2014 timepoint. The second reference is a WHO Global Antimicrobial Resistance Surveillance System report from 2022, that we consider complements the initial 2014 report from our current context. This is located underneath the ‘introduction’ section, as follows: “This is recognised by current international healthcare policy initiatives, as well as WHO AMR surveillance reports. The results of inter-continental surveillance has also underscored the importance of effective behavioural science-based research in this area.”

**6f) 'The recognition of social and behavioural sciences' perspectives has led to the development, evaluation, and implementation of effective psychological interventions aimed at reducing antibiotic prescribing within the context of AMR management' - references cited do not seem to support this claim. Costelloe (ref. 16) is not on the effectiveness of behavioural interventions. Hullscher (ref. 17) relates to hospitals, not primary care/community (which is the focus of this review). Atkins (ref. 18) introduces the Theoretical Domains Framework to investigate implementation problems and does not report an effective psychological intervention using behavioural sciences to reduce AMR. There are several relevant papers on this topic that could be referred to, e.g., [ref 3] , [ref 4] , [ref 5], [ref 6]**

 We thank the reviewer for this comment. Appropriate references for this sentence are now included within the manuscript, underneath the ‘introduction’ section, as follows: “The recognition of social and behavioural sciences’ perspectives has led to the development of psychological interventions targeting AMR in recent years.(22) Such interventions may be considered as BCIs, or “co-ordinated sets of activities designed to change specified behaviour patterns”(23)”

**6g) 'This framework (COM-B) has provided a theoretical lens for examining behaviour change interventions in primary care settings aimed at addressing antibiotic prescribing behaviours among providers' - the references cited do not seem to have used COM-B, certainly not explicitly. If the Authors are saying that we can better understand efficacious interventions by mapping the successful component(s) to COM-B, then that needs to be explained more clearly here. The way it is currently written implies the cited studies involved developing efficacious interventions using COM-B to address antibiotic prescribing.**

 We thank the reviewer for highlighting this point. We have now amended this sentence to enhance clarity for the purpose which we meant – “Prior studies indicate that this model can guide the analysis of psychological interventions aimed at improving antibiotic prescribing among HCPs”. The two references support this point – the first being a cross-sectional analysis of behaviour change interventions that influence antimicrobial prescribing and the second, a qualitative exploration in secondary care antimicrobial stewardship education and training implementation strategies. Both studies underpin their analysis of intervention and implementation strategies with the COM-B framework. This is located underneath the “introduction section”.

**6h) a randomised controlled trial (RCT) in the UK examined behaviour change approaches for reducing expectations for antibiotics in primary care' (ref. 28) - This description of the study is misleading. First, it was a randomised experiment, not a trial/RCT. Second, it involved hypothetical scenarios (different types of messages and their impact on intentions) and not actual behaviour.**

 We thank the reviewer for this point. On reflection, we acknowledge this is not an RCT in the conventional use of term in health services research and population health, therefore we will make the suggested distinction between a randomised experiment with this study. Revision of this sentence within the manuscript has occurred and now accurately reflects the contents of the cited paper. This is evident underneath the ‘introduction’ section, as follows: “With a focus on addressing ATSB, a randomised experiment in the UK examined behaviour change approaches for reducing hypothetical expectations for antibiotics in primary care.(30)”

**7) Review question and objectives**

**7a) The aim in this section differs from the one provided in the Abstract. The one provided in the abstract seems more suited to the choice of a scoping review.**

 We thank the reviewer for highlighting this point. This sentence has been revised to reflect matching aims in both the abstract and full-text. This is located underneath the ‘review question’ section, as follows: “This review aims to synthesise the extent and type of evidence on BCIs which address ATSB for RTIs in the primary and/or community care settings.

 The research questions forming the objectives of the review are:

- What BCIs have been applied and/or evaluated to reduce ATSB for RTIs?
- What attributes of BCIs can be characterised by coding and mapping them to the COM-B model within the ‘Behaviour Change Wheel’ framework?(23)
- What behaviour change theories are embedded within interventions that are used to reduce ATSB for RTIs, if any?
- What gaps exist presently in the literature that need to be addressed in future research for BCIs?”

**7b) Treatment seeking behaviour is mentioned in the abstract, whereas expectations for antibiotics are mentioned in the main text - these are different things. Do the Authors mean that they aim to identify BCIs that seek to lower patient/carer expectations for antibiotic prescription for RTI symptoms? The key concepts and focus of the review should be introduced in the Introduction (as per comment 5b) and then kept consistent in the Review aims and objectives.**

 We thank the reviewer for mentioning this key point. While we acknowledge that terminology and concepts are sometimes inconsistently used or specified in the field of behaviour change and antimicrobial resistance, we believe that ATSB encapsulates both patient demand and antibiotic expectation effectively.

 As per previous amendment, definitions accompanying “antibiotic treatment seeking behaviour”, as well as other key concepts, are provided within the introduction. We have amended the manuscript to be reflective of this throughout each manuscript section where the term had previously consisted within.

**7c) Do the Authors aim to map the intervention components to the COM-B model? What is the purpose of this and how is this objective suited to the choice of a scoping review (rather than a systematic review)?**

 We thank the reviewer for making this point. Our aim is to map and categorise the intervention components rather than evaluate the effectiveness of specific interventions themselves. A scoping review was recognised by the research team as suitable because it allows for a broad exploration and categorisation of existing interventions, identifying gaps and providing insights for future research, without the narrow focus on effectiveness typically seen in systematic reviews and other literature synthesis **.**

 Munn, Z., Pollock, D., Khalil, H., Alexander, L., Mclnerney, P., Godfrey, C.M., Peters, M. and Tricco, A.C., 2022. What are scoping reviews? Providing a formal definition of scoping reviews as a type of evidence synthesis. JBI evidence synthesis, 20(4), pp.950-952.

 Munn Z, Pollock D, Khalil H, Alexander L, Mclnerney P, Godfrey CM, Peters M, Tricco AC. What are scoping reviews? Providing a formal definition of scoping reviews as a type of evidence synthesis. JBI evidence synthesis. 2022 Apr 1;20(4):950-2.

**8) 'As diagnosed by an ICD-10 criterion' - Can the Authors explain what the ICD-10 criterion is? Is the review aim a relevant place to include this in? (It seems to be something that might be better explained in the Introduction.) It is also unclear what this phrase is meant to indicate - it is needed at all? Patient expectations for antibiotics are likely to be influenced by their (RTI) symptoms, not a diagnosis.**

 We thank the reviewer for highlighting this point. On reflection, we have now included mention of ICD-10 criteria within the ‘introduction’ section, as follows: “This in turn may impact coding system reports, such as those offered by the ICD-10 criteria for RTIs.” We have also taken note of your suggestion and adopted the symptoms listed within the ICD-10 criterion as a reference point for what an RTI stipulates, and will use this, as opposed to an official ICD-10 diagnosis necessary for study inclusion. We have specified this under the ‘participants’ section, as follows: “Eligible studies will focus on patients or carers within the primary and/or community care setting, that present with or discuss symptom(s) that typically constitute an RTI, as defined by ICD-10 RTI criteria.”

**9) Eligibility criteria**

**'Conceptually present with an RTI' - It is unclear what 'conceptually present' means. Perhaps it would be clearer to state instead 'present with RTI symptoms'.**

 We thank the reviewer for raising this point. On your suggestion, we have amended the manuscript to reflect “that present with or discuss symptom(s) that typically constitute an RTI, as defined by ICD-10 RTI criteria”, underneath the ‘participants’ section.

**10) 'As diagnosed by an ICD-10 criterion' - How is the ICD-10 operationalised here? What do the Authors mean by 'as diagnosed'? Is 'as diagnosed' an appropriate phrase here? (It suggests that ICD-10 diagnoses an RTI.)**

 We thank the reviewer for raising this point. Please see our response to your previous (9 ^th^) comment for the amendments we have made on this point.

**11)Concept - Are consultation and help/advice seeking behaviours included here? E.g. whether patients seek consultations for RTI symptoms, which may or may not involve/imply seeking, expecting or demanding antibiotics. (This links with our previous concepts around clarifying the concepts and focus of this review).**

 We thank the reviewer for raising this point. As described in the response 7b, we have now modified the manuscript to reflect accompanying definitions with key concepts including patient expectations, antibiotic demand, treatment seeking behaviours and supply-side solutions. We have now amended the manuscript to reflect the inclusion of consultation and help/advice seeking behaviours

**12) Search strategy**

**Inclusion of all languages - How will research in any language be read and data extracted? Does the review team have sufficient resources to do so?**

 We thank the reviewer for making this point. Please see our reply to reviewer 1’s third response for the amendments we have now made.

**13) 'Given the timing at which the implementation of the theoretical domains framework took place across interventions' - What do the Authors mean by this? Interventions might have been developed and tested regardless of the theoretical frameworks. In fact, interventions are often developed without the use of theory or frameworks. Moreover, which theoretical frameworks do the Authors refer to? There are many health behaviour change theories developed before 2000, and the Behaviour Change Wheel was published in 2014.**

 We thank the reviewer for making this point. Please see our reply to reviewer 1’s fourth response for the amendments we have now made.

**14) Linked with the previous comment, the rationale provided for applying a time limit is unconvincing. While we understand that time limits might be necessary, the reason for that should be clear (even if it is related to, e.g., feasibility, relevance of the expected literature etc.).**

 We thank the reviewer for making this point. Please see our reply to reviewer 1’s fourth response for the amendments we have now made.

**Methods**

**15) 'The proposed scoping review will be conducted in accordance with the JBI methodology for scoping reviews' - The reference provided for this is incorrect. The authors cite "The development of software to support multiple systematic review types: the Joanna Briggs Institute System for the Unified Management, Assessment and Review of Information (JBI SUMARI)" which is an article detailing the development of a software program to support systematic reviews not the JBI guidance/manual for conducting scoping review.**

 We thank the reviewer for highlighting this point. Appropriate references for this sentence are now included within the manuscript. Please see the amendment of this underneath the ‘method’ section, as follows: “The proposed scoping review will be conducted in accordance with the JBI methodology for scoping reviews.”

**16) 'JBI System for unified management‚Ä¶' - Reference for this is incorrect - currently number 25 which relates to 'Antimicrobial resistance: risk associated with antibiotic overuse and initiatives to reduce the problem' - presumably the Authors intended to cite the reference miscited above (point 15) pertaining to JBI SUMARI.**

 We thank the reviewer for highlighting this point. Appropriate references for this sentence are now included within the manuscript. Please see the amendment of this underneath the ‘method’ section.

**17) In line with the most recent recommendations for scoping review methodology (see Peters et al. (2022) 'Best practice guidelines and reporting items for the development of scoping review protocols'), we suggest the Authors begin the methods section by clearly identifying and correctly referencing the scoping review methodology/framework they have used (e.g., JBI, Arksey & O'Malley, Levac et al), and then throughout the protocol, the methodology/framework should be followed and sufficiently reported.**

 We thank the reviewer for highlighting this point. We have now amended the methods section to more accurately reflect that methodology that we plan to follow and report to i.e. JBI.

**Study/source of evidence selection**

**18) Why do the Authors propose to use both Covidence and JBI SUMARI if both systems can be used for all steps of the review process?**

 We thank the reviewer for highlighting this point. As JBI SUMARI was added in error, it has now been removed. Please see the amendment of this underneath the ‘study/source of evidence selection’ section, as follows: “Following the search, all identified citations will be collated and uploaded into Covidence®, with associated duplicates removed.”

**19) 'Titles and abstracts will be screened by two or more independent reviewers' - Does this mean that each title/abstract will be screened by at least two (possibly more) reviewers (i.e. duplicate screening)?**

 We thank the reviewer for highlighting this point. To confirm, the statement that "titles and abstracts will be screened by two or more independent reviewers" does indicate that each title and abstract will be screened by at least two reviewers, which constitutes duplicate screening. We have now amended the wording of this to enhance clarity for future reviews and to aid replicability. We also acknowledge the limitations of this, however, this is due to pragmatic reasons, as is the case in similar reviews of this nature. Please see the amendment of this underneath the ‘study/source of evidence selection’ section, as follows: “A minimum of 10% of full-texts from the selected studies will be evaluated against the inclusion criteria by two or more independent reviewers. The primary author will review all 100% of full-texts.”

 Peters MD, Godfrey CM, Khalil H, McInerney P, Parker D, Soares CB. Guidance for conducting systematic scoping reviews. JBI Evidence Implementation. 2015 Sep 1;13(3):141-6.

**Data extraction**

**20) Will the data extraction be conducted in duplicate? Other than the '10% of included papers', will the remaining data extraction be done by a single reviewer?**

 Please see our fourth response to reviewer 1’s comments.

**21) Table 1:**

**Population - suggest reporting also sample sizes;**

**Concept - type of BCI - how will it be determined? What is meant by 'to inform theoretical framework'?**

 We thank the reviewer for highlighting this point. We have now amended Table 1 “population” to report sample sizes. The determination of the type of behaviour change interventions will be guided by several factors, including intervention components, theoretical underpinnings (if any), as well as familiarisation with the literature. On reflection we acknowledge that ‘to inform theoretical framework' is potentially confusing, therefore we have modified this section to “theoretical basis”, which will be the behaviour change theory, if used/specified or reported.

**Data analysis**

**22) BCIs could be coded using the full Behaviour Change Wheel model, not only COM-B; e.g., intervention functions are useful and relevant. (However, it remains unclear to us how exactly this fits with the aim of the scoping review stated in the Abstract of synthesising the extent and type of evidence - as per comments 7a and 7c).**

 We thank the reviewer for highlighting this point. By coding BCIs with the COM-B model, we hope to provide an overview of the range of the content of interventions  which directly contributes to the review's aim of mapping the landscape of evidence.  This insight is critical for evaluating the current state of evidence and identifying gaps in the literature that warrant further exploration. Utilising the COM-B model allows the review to pinpoint areas where the evidence is lacking or where particular intervention functions have not been adequately tested. This identification of knowledge gaps can guide future research priorities and intervention development.

**23) How exactly will the COM-B model (concepts) be used in the analysis of the interventions? Will the intervention descriptions be coded using these theoretical concepts? We would suggest that a codebook and coding instructions are developed, with definitions of concepts (e.g. COM-B, intervention functions - for example from the Behaviour Change Wheel book) to ensure a correct and reliable coding process.**

 We thank the reviewer for highlighting this point. The intervention descriptions will be coded using the theoretical concepts from the COM-B model. This process will involve a comprehensive codebook, detailing how each of the COM-B components is defined and operationalised. This will include specific criteria for identifying interventions that enhance capability, opportunity, or motivation. Clear coding instructions are now mentioned, to ensure consistency among reviewers. This includes guidelines on how to categorise each intervention based on its description. Please see the amendment of this underneath the ‘appendix’ section.

**Minor points:**

**Introduction - it should be 'an increasing trend of sporadic‚Ä¶'**

 We thank the reviewer for highlighting this point. We have now rectified and removed this sentence in adjusting other sentences based on previous comments made.

**'Behaviour change in this domain' - 'domain' is an odd word choice; perhaps a more straightforward word (e.g. 'area') would be clearer**

 We thank the reviewer for highlighting this point. We have now rectified the sentence to specify “area” instead of “domain”. This amendment is listed underneath the ‘introduction’ section, as follows: “Behaviour change interventions (BCIs), in this area, can be seen as one segment of a collective, proactive approach in promoting prudent antibiotic use for respiratory tract infections (RTIs) amongst primary care networks and within community care settings.(5)”

**'Compared to the preceding two years, namely 2020 and 2019' - the sentence establishes this as '2022 data' so by stating the comparison is with the preceding two years means the inclusion of 'namely 2020 and 2019' is redundant.**

 We thank the reviewer for highlighting this point. Through revision of the manuscript, the sentence that this point relates to is now omitted.

**Abbreviation RTIs should be used consistently after being introduced, e.g. 'up to 60% of cases of acute respiratory infections.'**

 We thank the reviewer for highlighting this point. Through revision of the manuscript, the sentence that this point relates to is now omitted.

**The last sentence of the Introduction: a comma in 'aimed, to' should be removed; suggest revising the sentence as it repeats 'aimed' and is quite long and complex.**

 We thank the reviewer for highlighting this point. This sentence has now been amended to be grammatically correct and more coherent. This amendment is listed underneath the ‘introduction’ section, as follows: “To our knowledge, there is no other systematic or scoping review that looks to synthesise the available evidence on BCIs aimed at patients within primary and/or community care settings, to reduce antibiotic treatment seeking behaviour for RTIs.”

**The sub-heading is 'review question' but the section does not provide questions - it reports the aim and objectives.**

 We thank the reviewer for highlighting this point. Questions are now reported under the sub-heading “review question”. This amendment is listed underneath the ‘review question’ as follows: “The research questions forming the objectives of the review are:

- What BCIs have been applied and/or evaluated to reduce ATSB for RTIs?
- What attributes of BCIs can be characterised by coding and mapping them to the COM-B model within the ‘Behaviour Change Wheel’ framework?
- What behaviour change theories are embedded within interventions that are used to reduce ATSB for RTIs, if any?
- What gaps exist presently in the literature that need to be addressed in future research for BCIs?”

**Review questions - Why is this section (and some subsequent sections) in italics?**

 We thank the reviewer for highlighting this point. This formatting other is now rectified within the manuscript.

**Eligibility criteria: should be 'focus on the patients or carers', not patient.**

 We thank the reviewer for highlighting this point. We have now amended this sentence to accurately reflect the focus on patients or carers. This amendment is listed underneath the ‘eligibility criteria’ section, as follows: “Eligible studies will focus on patients or carers within the primary and/or community care setting, that present with or discuss symptom(s) that typically constitute an RTI, as defined by ICD-10 RTI criteria. ^”^

**Context - 'focuses' should be 'focus' as refers to publications; no comma needed.**

 We thank the reviewer for highlighting this point. We have now amended this sentence to be grammatically correct. This amendment is listed underneath the ‘context’ section, as follows: “Relevant publications in the English language from any country that focus on primary and/or community care will be included.”

**'One independent reviewer will complete remaining screening' - should be just 'one reviewer'; being 'independent' only is relevant if multiple people double screen the same records.**

 We thank the reviewer for highlighting this point. We have now rectified this sentence and excluded “independent” as you rightly deemed was irrelevant. This amendment is listed under the ‘data extraction’ section, as follows: “The primary author will review all 100% of full-texts.”

# Reviewer report: Reviewer response for version 1

**DOI:** 10.21956/hrbopenres.15151.r42210

**Published:** 2024-09-10

**Authors:** Regina Poß-Doering

**Affiliations:** 1 Department of General Practice and Health Services Research, University Hospital Heidelberg, Heidelberg, Germany

This is a study protocol for a first scoping review aiming  to synthesize extent and type of behavior change interventions which address antibiotic treatment behavior for respiratory tract infections in the primary care setting.

 Description of methods to be used in the proposed review is transparent and it is to be assumed that the study would be replicable. 

 Authors should attend to the following aspects:

 - It is intended to include studies published since 2000, given the timing at which the implementation of the theoretical domain’s framework took place across interventions. There is no description of the TDF and why it is chosen as a refernce point.  

 - It appears to be unclear how unpublished studies could be included in the review.

 - It is not transparent to what extent the search strategy might be adapted for each database. 

 -  It is also not transparent how studies in any language can be assessed for inclusion or exclusion. How many languages can realsitically be covered by the reasearch team?

 -  Data will be extracted from at least 10% of included papers: What is the rationale here, and what about the remaining 90%?

 My recommendation would be to address these aspects in the protocol.   

Is the study design appropriate for the research question?

Yes

Is the rationale for, and objectives of, the study clearly described?

Yes

Are sufficient details of the methods provided to allow replication by others?

Yes

Are the datasets clearly presented in a useable and accessible format?

Not applicable

Reviewer Expertise:

health services research; implementation science; process evaluations; rational use of antibiotics; holistic chronic non-cancer pain case and care management (in primary care)

I confirm that I have read this submission and believe that I have an appropriate level of expertise to confirm that it is of an acceptable scientific standard, however I have significant reservations, as outlined above.

# Response

**Published:** 2024-12-09

**Authors:** Anthony Maher

**Affiliations:** School of Psychology, University of Galway, Galway, County Galway, Ireland

Many thanks for your positive comments and insightful feedback. We greatly appreciate your time in reviewing our protocol. We have updated the protocol based on your comments. In particular, we have tried to enhance the clarity of our methods section, to further future replicability of this study type.

***It is intended to include studies published since 2000, given the timing at which the implementation of the theoretical domain’s framework took place across interventions. There is no description of the TDF and why it is chosen as a refernce point.***

 We thank the reviewer for identifying this weakness in our rationale for the time limit. As both reviewers identified this issue, we have reflected and have simplified the main rationale for selecting this time range/limit. The primary determinant was a pragmatic decision based on time and resources available to conduct this review. We also feel that this focus on the last two decades will more accurately capture contemporary literature and healthcare practice. Please see underneath the ‘search strategy’ section, as follows: “Only studies published since 2000 will be included for pragmatic reasons.”

***It appears to be unclear how unpublished studies could be included in the review.***

 We thank the reviewer for highlighting this point. Protocols for the inclusion of grey literature including unpublished studies are now specified within the study, underneath the ‘search strategy’, as follows: “The text words contained in the titles and abstracts of relevant articles, and the index terms used to describe the articles will be used to develop a full search strategy for Medline, Embase, CINAHL, PsycINFO, Web of Science Core Collection, Scopus, EThOS and Google Scholar (see Appendix I).” The inclusion criteria for unpublished studies will be the same for those that are published.

***It is not transparent to what extent the search strategy might be adapted for each database. It is also not transparent how studies in any language can be assessed for inclusion or exclusion. How many languages can realistically be covered by the research team?***

 We thank for the reviewer for raising this point. As the review methodology has evolved in recent months, we acknowledge the lack of feasibility of having translation of all non-English material. We now specify that only English language publications will be included in the review. This is specified underneath the ‘search strategy’ section, as follows: “The search strategy, including all identified keywords and index terms, will be adapted for each included database and/or information source. The reference list of all included sources of evidence will be screened for additional studies. Studies published in the English language will be search for and included.” We will acknowledge the limitations of this in our discussion, once the study has been performed.

***Data will be extracted from at least 10% of included papers: What is the rationale here, and what about the remaining 90%?***

 We thank the reviewer for highlighting this point. As stated, 10% of the included papers will undergo a second review of the extracted data by an independent reviewer to ensure accuracy. For the remaining studies, data extraction will be performed by a single reviewer. This approach hopes to balance efficiency with the need for accuracy, allowing the research team to manage their resources effectively while still maintaining a level of oversight on the data extraction process. Conducting duplicate data extraction for all studies is considered by the research team resource-intensive and time-consuming, especially in a scoping review where a large volume of literature is often assessed. This will be reflected in the finished manuscripts limitations.
